# Timing of internal processes: Investigating introspection about the costs of task switching and memory search

**DOI:** 10.3758/s13414-022-02510-6

**Published:** 2022-05-24

**Authors:** Daniel Bratzke, Donna Bryce

**Affiliations:** 1 Department of Psychology, University of Bremen, Bremen, Germany; 2 Department of Psychology, Eberhard Karls University of Tübingen, Tübingen, Germany

**Keywords:** Task switching, Memory search, Introspection, Awareness

## Abstract

During the last two decades, there has been new interest in introspection about multitasking performance. In this field, subjective timing of one’s own reaction times (introspective RTs) has proven a useful measure to assess introspection. However, whether timing our own cognitive processing makes use of the same timing mechanisms as timing external intervals has been called into question. Here we take a novel approach to this question and build on the previously observed dissociation between the interference of task switching and memory search with a concurrent time production task whereby temporal productions increased with increasing memory set size but were not affected by switch costs. We tested whether a similar dissociation could be observed in this paradigm when participants provide introspective RTs instead of concurrent temporal productions. The results showed no such dissociation as switch costs and the effect of memory set size on RTs were both reflected in introspective RTs. These findings indicate that the underlying timing mechanisms differ between temporal productions and introspective RTs in this multitasking context, and that introspective RTs are still strikingly accurate estimates of objective RTs.

## Introduction

Numerous studies have now demonstrated that introspection about one’s own multitasking performance can be severely distorted (Bratzke & Bryce, 2016; Bratzke & Janczyk, 2021; Bryce & Bratzke, 2014, 2015, 2017; Bratzke et al., 2014; Corallo et al., 2008; Marti et al., 2010). These studies all used a classical dual-task paradigm, the Psychological Refractory Period (PRP) paradigm (see Pashler, 1994). In this paradigm, two tasks are presented with different temporal overlap (i.e., with different stimulus onset asynchronies, SOAs) and participants are asked to provide separate speeded responses for the two tasks. What is usually observed in this paradigm is an increase of reaction time (RT) for the second task with decreasing SOA, the PRP effect. Most of the previous introspective studies on this effect used visual analogue scales (VASs) to assess estimates of RT (introspective reaction times, IRTs) after each trial. They observed that participants were apparently unaware of their dual-task costs, as the PRP effect was not reflected in IRTs. Notably, concurrent dual-tasking constitutes only one pole of the multitasking continuum (see Salvucci et al., 2009), and only one study so far has investigated introspection about multitasking performance at the other pole, that is, when people switch between tasks without temporal overlap between the tasks. Here, the evidence differs from the concurrent dual-task situation in the PRP paradigm, as switch costs were reflected in IRTs (Bratzke & Bryce, 2019).

We have previously provided evidence that introspection about RT performance in dual-task contexts differs from timing external intervals (Bryce & Bratzke, 2017) and that other temporal intervals in a trial can influence IRTs (e.g., Bratzke & Bryce, 2019). As such, in some cases introspection about RT performance seems to rely on retrospective rather than prospective timing mechanisms (e.g., Bratzke & Bryce, 2016, 2019; see also Klein & Stolz, 2018). It is often assumed that prospective and retrospective timing are based on different types of information (e.g., Zakay & Block, 2004; but see, e.g., Brown, 1985). According to this view, prospective timing (also called “experienced time”) requires attentional resources and the information used for timing is time-based (e.g., pulses elicited by a pacemaker). In contrast, retrospective timing (also called “remembered time”) is less attentionally demanding (because there is no attention devoted to time) and the information used is memory-based (e.g., the number of events or contextual changes during a time interval). This distinction shares many commonalities with a distinction from the field of metacognition regarding the processes involved in metacognitive monitoring. The direct access account of monitoring (e.g., Nelson & Narens, 1990) describes a privileged, direct view of our task performance that may be attentionally demanding, while the cue-utilization account of monitoring (e.g., Koriat, 2012) describes the selection and integration of various cues that may be more or less valid predictors of task performance.

In introspective multitasking experiments, participants know in advance that they should provide an estimate of their RT at the end of each trial, and as such providing IRTs is a prospective timing task. However, that participants know in advance about the timing task does not necessarily mean that the timing mechanism involved is prospective in nature. In fact, we have evidence from the dual-task context that IRTs oftentimes rely more on retrospective than on prospective timing mechanisms. This is essentially for three reasons. First, introspective measures of RT can be strongly biased by non-temporal as well as temporal cues other than RT, such as the feeling of difficulty (Bryce & Bratzke, 2014), the cue-stimulus interval and the response-stimulus interval in task switching (Bratzke & Bryce, 2019), and the distribution of comparison intervals in a dual-task paradigm where participants had to compare their RT with comparison intervals (Bratzke & Bryce, 2016). Second, previous results from IRT studies are quite consistent with respect to effects of task difficulty on IRT showing the signature of retrospective timing (higher task difficulty prolongs perceived time) rather than prospective timing (higher task difficulty shortens perceived time; for evidence regarding these signatures, see, e.g., Zakay & Block, 2004). Third, in situations with high attentional demands (as, e.g., in multitasking) spare resources for timing may be rather low, making prospective estimates impossible and prompting estimates that are rather retrospective in nature (cf. Zakay & Block, 2004).

In the present study, our main aim was to gain further insights into the nature of IRTs in the task-switching context by investigating whether the timing processes involved in producing IRTs are the same as those involved in timing an external interval. The experimental paradigm built on a series of experiments reported by Fortin et al. (2010; see also Viau-Quesnel & Fortin, 2014). In these experiments, participants performed a task-switching paradigm with a memory search (M) task and a digit classification (C) task. Each trial consisted of an initial phase in which a set of letters was presented, followed by a sequence of two tasks with either a repetition of the task (MM or CC) or a switch between the tasks (MC or CM). One group of participants provided speeded responses to the two tasks, as is usually the case in such paradigms. Another group of participants had to postpone their response to the second task until they judged 2 s had passed (starting 400 ms before Task 2 presentation). The results of the speeded-RT group showed the standard effects of task switching and memory set size on RT. The results of the time-production group, however, showed a differential pattern: Time productions were affected by memory set size, but not by task switching. According to Fortin et al. (2010), these results suggest that memory search interrupts the accumulation of temporal information during timing whereas task switching does not.

In the present study, we reasoned that if introspective RTs rely on the same timing mechanisms as the temporal productions in the studies by Fortin and colleagues, a dissociation between switch costs and memory search costs should also be observed with IRTs. Since we already had observed that switch costs can be reflected in IRTs (Bratzke & Bryce, 2019), this dissociation would most likely involve no effect of memory search on IRTs. This would also be consistent with Fortin et al.’s suggestion that the accumulation of temporal information is interrupted during memory search. Accordingly, we replicated the experimental procedure by Fortin et al. (2010) but replaced the temporal production with an explicit report of IRT2. Since the effects of experimental manipulations can be directly assessed with this design, it was not necessary to employ a second group of participants who only performed the speeded RT tasks as in Fortin et al.’s studies. An additional aim of our study was to establish what information contributes to IRTs in this multitasking context. We addressed this via a linear mixed effect model using IRT as the dependent variable and task sequence, memory set size, and RT as predictors (see also Bratzke & Bryce, 2019). We assume that if timing of our own cognitive processes in task switching is prospective in nature (namely, “time-based”), RT should be the main predictor of IRT, whereas if it is retrospective in nature, other predictors (i.e., task sequence and memory set size) should also contribute to IRT. If timing is interrupted by memory search, there may still be a positive relationship between RT and IRT, but set size should be a significant negative predictor of IRT.

## Method

### Participants

Twenty students of the University of Tübingen participated for monetary compensation or course credit (mean age = 22.1 years; 18 female). This sample size was chosen to approximately double the sample size of previous experiments (the size of the temporal production groups in Fortin et al., 2010, varied between *n* = 7 and *n* = 12). All participants reported normal or corrected-to-normal vision, were naïve regarding the underlying hypotheses, and provided written informed consent prior to data collection.

### Apparatus and stimuli

The experiment was run in a sound-attenuated, dimly illuminated experimental booth. The experiment was programmed in Matlab using the Psychophysics Toolbox extension (Brainard, 1997; Pelli, 1997) version 3.0.8. Participants sat in front of an iMac computer (OS X; built-in 60-Hz monitor) with a viewing distance of approximately 50 cm.

All stimuli were presented in white against a black background. All consonants of the alphabet (capital letters) served as stimuli for the memory task, and the digits *2* to *9* as stimuli for the digit classification task. Letters and digits were presented in Arial (font size 24 pt). The letters for the memory set (2 vs. 6) were randomly drawn from all possible letters. In the memory task, a letter was randomly drawn from the memory set in “present” trials, and from the set of remaining letters in “absent” trials. In the digit classification task, an odd (even) number was randomly drawn from the set of odd (even) numbers. In non-switch trials, there was the additional restriction that S1 could not be repeated as S2. The A and the L keys of a standard German keyboard were used to record responses with the left and right index finger. The visual analogue scale (VAS) for collection of IRTs was marked every 250 ms and labelled at the left (0 s) and the right end (2 s). A mouse was used to provide IRTs.

### Tasks and procedure

At the beginning of each trial, a fixation screen (*****) was presented and the participant pressed the space bar when they were ready to view the memory items. After the key press, a stream of letters (2 vs. 6) was presented, each for a duration of 1 s. Then the question “Bereit?” (German for “Ready?”) appeared on the screen until the participant again pressed the space bar. After a blank screen of 500 ms, the stimulus for Task 1 (digit or letter) was presented until the participant pressed one of the response keys. After another blank interval of 200 ms, the stimulus for Task 2 (digit or letter) was presented. Immediately after the participant’s response, a VAS appeared on the screen. Participants provided IRTs by clicking with the mouse on the VAS. A small dot appeared with the mouse click at the respective position confirming the participant’s estimate (clicking on a position outside of the VAS was not possible). The next trial started after a blank intertrial interval of 500 ms.

**Fig. 1:**
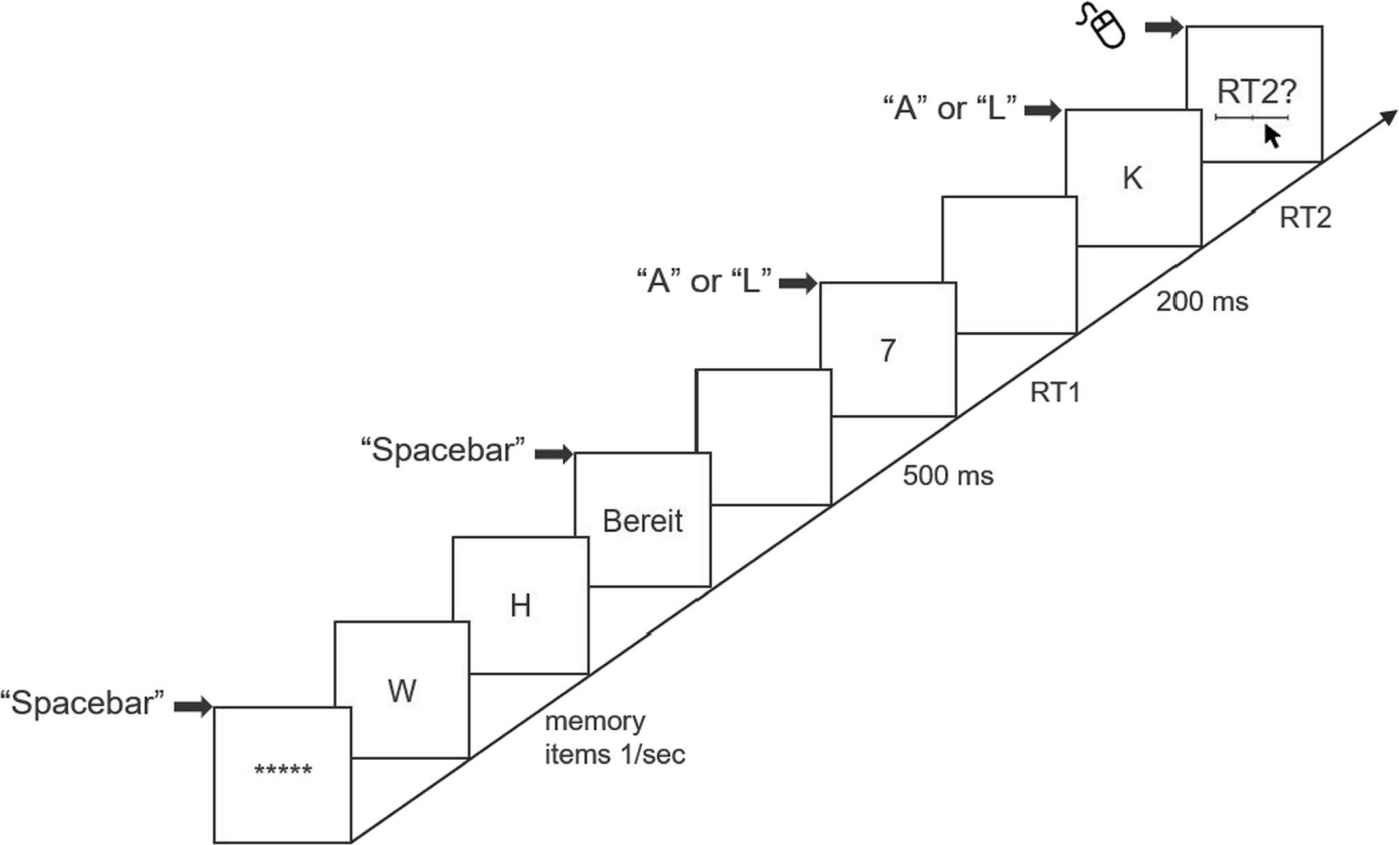
Procedure of an experimental trial. At the beginning of each trial, two or six memory items (consonants, 1/s) were presented. Then, a sequence of two tasks was presented, which could be either the same task or different tasks (memory search vs. digit classification). In the depicted example, Task 1 is a digit classification task and Task 2 is a memory search task. In the digit classification task, participants had to indicate with a keypress whether a digit (2–9) was even (“A”) or odd (“L”). In the memory search task, a consonant was presented and participants had to indicate whether the stimulus had been present (“A”) in the memory set or not (“L”). At the end of each trial, a visual analogue scale was presented and participants were asked to provide an estimate of their reaction time in the second task by clicking with the mouse on the scale

In the memory task, participants were instructed to provide a keypress with the left (right) index finger if the presented letter was present (absent) in the memory set. In the digit classification task, participants were required to provide a keypress with the left (right) index finger if the digit was odd (even). Instructions emphasized speed and accuracy in the RT tasks. For IRT collection, participants were explicitly instructed (written and oral instruction) to estimate the interval between the onset of S2 (letter or digit) and their response to S2. For the IRT assessment, there were no time restrictions (Fig. 1).

All combinations of possible task sequences (MM, MC, CM, CC), odd/even digits in the digit classification task and present/absent trials in the memory search task were tested equally often. Combined with two levels of memory set sizes (2 vs. 6), this resulted in 32 unique trials, which formed an experimental block. In total, there were eight experimental blocks and one initial practice block with 16 trials (randomly drawn from all possible trials).

## Results

The main data analysis was similar as in Fortin et al. (2010). Trials with errors in at least one of the two tasks were removed from RT analysis (Task 1: 8.2%; Task 2: 9.8%; 16.5% in total). In a next step, trials with RT1, RT2, and/or IRT2 deviating more than 3 *SD*s from the individual mean were removed from RT analysis (3.9% of correct trials). Separate ANOVAs with the within-subjects factors task sequence (switch vs. non-switch) and memory set size (2 vs. 6) were performed on RT2, IRT2, and error rate in Task 2. In addition to this analysis, we performed a linear mixed effect (LME) model analysis to investigate which variables (of task sequence, memory set size, and RT2) contributed to IRTs (see also Bratzke & Bryce, 2019). For this analysis, unaggregated RT2s and IRT2s were z-transformed. The fitted model included all main effects and interactions in the fixed effect structure and random slopes and intercepts for RT2 per participant. The R package LME4 (Bates et al., 2015) was used to fit LME models using REML, and *p*-values were derived using the Satterthwaite approximation (R package LmerTest; Kuznetsova et al., 2017; see also Luke, 2017). For all figures, standard errors for within-subject designs were calculated according to Morey (2008).

**Fig. 2:**
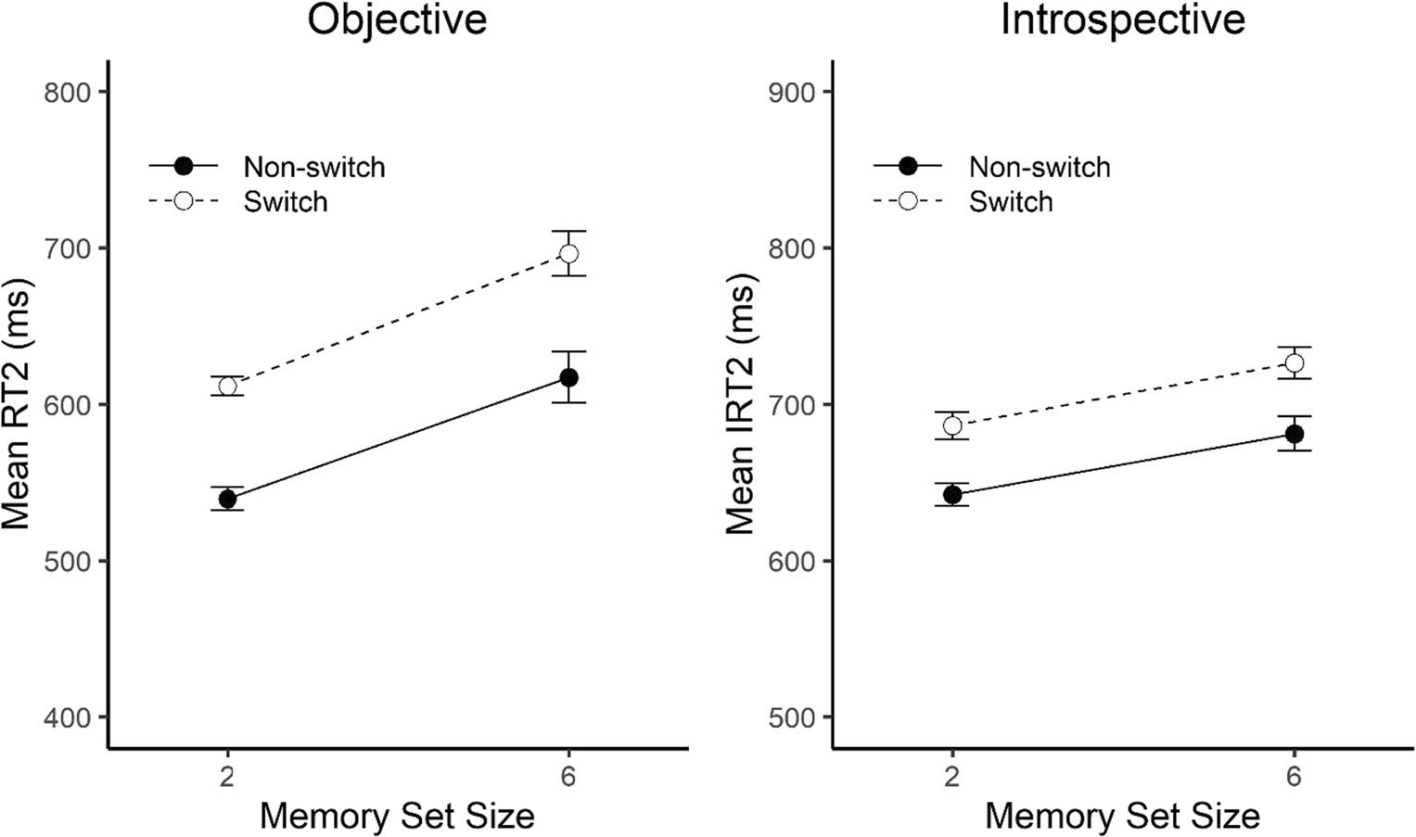
Mean reaction time (RT) and mean introspective reaction time (IRT) in Task 2 as a function of task sequence and memory set size. Error bars represent ± 1 within-subjects *SE*

Figure 2 shows RT2 and IRT2 as a function of task sequence and memory set size. As in Fortin et al. (2010), the ANOVA on RT2 revealed significant main effects of task sequence and memory set size. There were switch costs of 76 ms, *F*(1, 19) = 122.17, *p* < .001, η_p_^2^ = .87, and RT2 was on average 81 ms longer for the large compared to the small memory set, *F*(1, 19) = 78.97, *p* < .001, η_p_^2^ = .81. The interaction was not significant, *F*(1, 19) = 1.09, *p* = .310, η_p_^2^ = .05. The ANOVA on IRT2 revealed very similar results, as both main effects were significant. The effect of task sequence on IRT2 was 45 ms, *F*(1, 19) = 32.01, *p* < .001, η_p_^2^ = .63, and the effect of memory set size was 40 ms, *F*(1, 19) = 17.82, *p* < .001, η_p_^2^ = .48. Just as for RT2, there was no interaction between task sequence and memory set size on IRT2, *F*(1, 19) = 0.02, *p* = .885, η_p_^2^ < .01. The same overall pattern was also mirrored in error rates for Task 2. Participants made more errors in switch than in non-switch trials (11.6% vs. 8.0%), *F*(1, 19) = 7.24, *p* = .014, η_p_^2^ < .28, and also more errors in trials with the large compared to the small memory set size (12.1% vs. 7.4%), *F*(1, 19) = 19.40 , *p* < .001, η_p_^2^ = .51. Again, there was no significant interaction effect, *F*(1, 19) = 0.16 , *p* = .695, η_p_^2^ = .01.

**Fig. 3:**
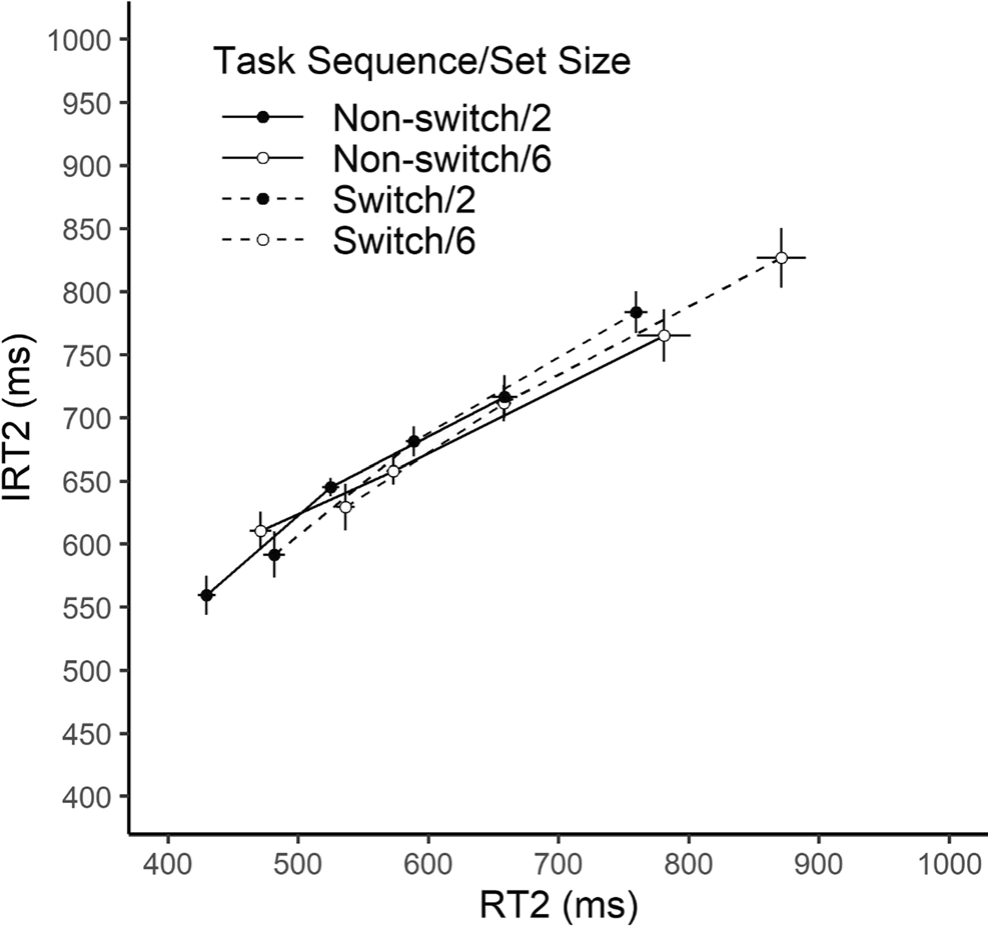
The relationship between objective and introspective reaction time in Task 2 (RT2 and IRT2). IRT2 is plotted against RT2 (divided into three bins, vincentized) as a function of task sequence and memory set size. Error bars represent ±1 within-subject *SE*

Figure 3 shows the relationship between IRT2 and RT2 (divided into three bins) as a function of task sequence and memory set size. Overall, a clear positive relationship between RT2 and IRT2 can be seen. Moreover, all IRT2-RT2 functions seem to have similar slopes and largely lie on top of each other. This observation was confirmed by the LME model analysis, which indicated only a significant effect of RT2 on IRT2, β = 0.38, *t*(24.9) = 6.77, *p* < .001 (all other *p*s ≥ .268).^1^

## Discussion

The present study assessed how participants introspect about their RT performance in a task-switching paradigm with a memory search task and a digit classification task. Switch costs and the effect of memory set size were both reflected in subjective estimates of RT (IRTs). Thus, participants were able to report the time demands of task switching and memory search. An additional in-depth analysis of the trial-wise IRT-RT relationship showed that there was hardly any influence of the factors task sequence and memory set size on IRTs beyond the positive IRT-RT relationship. Thus, participants seemed to be equally as good in judging their own RTs regardless of whether the trial involved a switch or a larger memory set size.

The present results did not show the dissociation regarding interference between timing and switch costs on one hand and the costs of memory search on the other hand, reported by Fortin and colleagues (Fortin et al., 2010; Viau-Quesnel & Fortin, 2014). In their experiments, which shared the basic design of the present study, participants performed a temporal production concurrently with Task 2 processing. As a result, temporal productions increased with increasing memory set size but were unaffected by switch costs. Since we did not observe the same dissociation in the present experiment, the timing mechanism involved in providing IRTs seems to differ from the one involved in concurrent temporal production. Of course, one limitation regarding this conclusion is that it is based on the comparison between different studies as the temporal production task of Fortin et al. was not included in the present study. However, we believe that such a comparison is justified because these authors observed the dissociation between memory set size and switch costs across two studies and four experiments and the present study was very similar to these previous experiments, with the exception that participants provided IRTs instead of temporal productions.

Another limitation of the present study is that the timing methods used in the present (VAS) and Fortin et al.’s (temporal production) study were quite different with respect to the involved response (i.e., a mouse click on a VAS in the present case and a termination of a pre-learned interval with a key press in Fortin et al.’s study). A temporal reproduction task, in which participants provide their IRTs by terminating a reproduction interval with a keypress, would be more similar to the temporal reproduction task of Fortin et al. in this respect. However, we believe that it is very unlikely that this confound contributed to the differential effects because the assessment of IRTs via VAS and temporal reproduction have yielded very similar results in a previous introspective PRP study (Bryce & Bratzke, 2015).

Various interpretations can be drawn from these results, one of which is that IRTs are not time-based at all and rather retrospective timing is used to produce IRTs. In the present study, relevant non-temporal information could be the number of memory items and the knowledge about whether a given trial sequence involved a task switch or not. If participants based their IRTs on such non-temporal information one would expect, for example, a vertical shift of the IRT-RT function depending on whether a task switch occurred or not. In other words, participants would simply provide larger IRTs in switch compared to non-switch trials irrespective of their actual RT in these trials. In fact, our previous results provided evidence for such a bias beyond the positive IRT-RT relationship in two out of three experiments (Bratzke & Bryce, 2019). The present observation that the effects of task sequence and memory set size on IRTs can be solely attributed to the positive IRT-RT relationship (task sequence did not affect the IRT2-RT2 relationship in the trial-wise LME model, and memory set size affected the IRT2-RT2 relationship only when the analysis was restricted to the trials in which a memory search occurred; see Footnote 1) is inconsistent with the assumption that IRTs are completely retrospective in this context, that is, that they are exclusively based on non-temporal information.

Our results indicate that timing of internal cognitive processing is different from timing an external interval, but that timing of cognitive processing in task switching and memory search still seems predominantly prospective in nature (i.e., time-based) and strikingly accurate. Interestingly, Ruthruff and Pashler (2010) observed a similar dissociation between concurrent time production and noting a duration in order to give a temporal reproduction in a dual-task context. They argued that when attentional resources are occupied by another task, timing could be achieved by a qualitatively different timing mechanism, probably much more implicit in nature than the one used for explicit timing of external events. Klein and Stolz (2018) also provided evidence for a distinction between timing of internal and external intervals by demonstrating that timing of an externally defined interval (the presentation duration of a stimulus) interfered with a concurrent demanding non-temporal task (verification of mathematical equations) but the timing of an internally defined interval (the RT to each stimulus) did not. One reason for this distinction might be that during active task processing, internal processes provide a rich source of non-temporal (internal) information that can be used to derive an estimate of RT, a source of information that is not available to the same degree when timing an external interval (see also Klein & Stolz, 2018). Furthermore, the observation of a positive RT-IRT relationship does not prove that IRTs are solely based on time information because the relationship could be driven by other (introspectively accessible) variables like internal preparatory state, ease of processing, and mental effort.

Besides the apparent dissociation between the present IRT results and Fortin et al.’s previous temporal production results, the present results confirm our previous observation that participants can report their switch costs in a trial-by-trial manner (Bratzke & Bryce, 2019). This result also fits well with recent results from adaptive voluntary task switching studies (Mittelstädt et al., 2018, 2019; Monno et al., 2021). In these studies, the stimulus for a task repetition appeared with an SOA that increased with the number of task repetition. It was consistently observed that participants usually switch to the other task when the SOA corresponds to their switch costs, which suggests that participants can not only access their switch costs in this paradigm, but also (implicitly or explicitly) use this introspective knowledge for behavioral adaptations.

Our second result that participants could also report the time demands associated with memory search is largely consistent with a previous study by Reyes and Sackur (2018). These authors asked their participants for subjective estimates of the number of scanned items (SNSIs) after each trial of two different memory tasks (judgment of recency and item recognition). Similar to the correspondence between the effect of memory set size on objective and introspective RTs in the present study, their results showed a correspondence between the effects of memory set size on RT performance and SNSIs in both tasks. However, the authors additionally observed a dissociation between the two memory tasks with respect to the effect of target position on RT and SNSIs; target position affected RT in both memory tasks but affected SNSIs only in the judgment of recency task. The authors argue that this result pattern reflects differences in processing complexity between the two memory tasks, assuming a serial memory scanning in the judgment of recency task and a direct access mechanism in the item recognition task. Together with the present results this suggests that people’s introspection can access the complexity as well as the time demands of the memory search process. Nevertheless, it would be interesting to assess introspective RTs in an experiment similar to the one by Reyes and Sackur to further investigate the differential contribution of temporal processing demands and processing complexity to introspective RTs.

In conclusion, the present evidence suggests that the underlying timing mechanism of introspective RTs differs from the one involved in timing an external time interval, but that IRTs still show a strong trial-by-trial relationship with objective RTs, which is consistent with a prospective (i.e., time-based) nature of IRTs in this context. Furthermore, the present results add to the growing body of IRT results indicating that introspection is not blind to the costs of task switching and provide novel insights into introspection about the time demands of memory search. These findings indicate that introspections about one’s own speed of responding in attentionally demanding sequential multitasking contexts can be remarkably accurate.

## Data Availability

The data generated and analyzed during the current study are available on the Open Science Framework via this View-only link: https://osf.io/ygk96/?view_only=794e90f84697403c8511f4db725c4b4a. The data will be made freely accessible on publication.

## Footnotes

1 For the sake of comparability, in our main analyses we followed Fortin et al. (2010) and included all possible task sequences. With respect to the effects of memory set size on RT2 and IRT2 this may not be optimal because trials in which there was no memory task at all, or only a memory task in Task 1 are included. We therefore performed additional analyses only for trials in which Task 2 was a memory task. The results showed two deviations from the results of our main analysis. First, there was a significant interaction between task sequence and memory set size on RT2, *F*(1, 19) = 9.38 , *p* = .006, η_*p*_^2^ = .33, with larger switch costs for the small (96 ms) than for the large (64 ms) memory set. Although this pattern was descriptively reflected in IRT2 (67 ms vs. 54 ms), the interaction was not significant, *F*(1, 19) = 0.49, *p* = .491, η_*p*_^2^ = .03. Second, the LME model analysis revealed a significant effect of memory set size on IRT2, indicating the IRTs were relatively underestimated when the memory set was large compared to when it was small, *β* = -0.08, *t*(204.7) = 2.14, *p* = .032.

## Declarations

### Open Practices Statement

The data generated and/or analyzed during the current study are available here: https://osf.io/ygk96/?view_only=794e90f84697403c8511f4db725c4b4a. The experiment was not preregistered.
